# Defect structure transformation after thermal annealing in a surface layer of Zn-implanted Si(001) substrates

**DOI:** 10.1107/S0021889813010169

**Published:** 2013-06-07

**Authors:** Kirill Shcherbachev, Vladimir Privezentsev, Vaclav Kulikauskas, Vladimir Zatekin, Vladimir Saraykin

**Affiliations:** a Semiconductor and Dielectric Materials Science, National University of Science and Technology ‘MISIS’, Leninskii prospekt, Moscow, 119049, Russian Federation; b Institute of Physics and Technology RAS, Moscow, Russian Federation; c Skobeltsyn Institute of Nuclear Physics, Lomonosov Moscow State University, Moscow, Russian Federation; d Russian Research Institute of Physical Problems, Zelenograd, Moscow, Russian Federation

**Keywords:** X-ray diffraction, ion implantation, radiation-induced defects, silicon

## Abstract

A combination of high-resolution X-ray diffractometry, Rutherford back scattering spectroscopy and secondary-ion mass spectrometry (SIMS) methods were used to characterize structural transformations of the damaged layer in Si(001) substrates heavily doped by Zn ions after a multistage thermal treatment. The shape of the SIMS profiles for Zn atoms correlates with the crystal structure of the damaged layer and depends on the presence of the following factors influencing the mobility of Zn atoms: (i) an amorphous/crystalline (a/c) interface, (ii) end-of-range defects, which are located slightly deeper than the a/c interface; (iii) a surface area enriched by Si vacancies; and (iv) the chemical interaction of Zn with Si atoms, which leads to the formation of Zn-containing phases in the surface layer.

## Abstract 2

A combination of high-resolution X-ray diffractometry, Rutherford back scattering spectroscopy and secondary-ion mass spectrometry (SIMS) allowed the influence of structural transformations in the damaged layer of Zn-doped Si(001) substrates after a multistage thermal treatment to be revealed. The shape of the Zn SIMS profiles correlates with the crystal structure of the layer and depends on the presence of factors influencing the mobility of Zn atoms.

## 1. Introduction

Recently, the fundamental properties of metal–oxide nanoparticles (NPs) in dielectric matrices have been intensively investigated, since such materials can be used in modern electronic and optoelectronic devices. ZnO NPs play an important role among them, since ZnO has a wide direct band gap (3.37 eV), large exciton binding energy (60 meV), room-temperature ferromagnetism and a chemical-sensing effect. ZnO NPs on Si substrates can be made by ion implantation. Therefore, the study of properties of nanostructured materials becomes highly relevant. Here, we present a study of the structure transformation of a damaged layer during thermal annealing of Zn^+^-implanted Si by high-resolution X-ray diffraction (HRXRD) complemented with Rutherford back scattering spectroscopy (RBS) and secondary-ion mass spectrometry (SIMS) techniques.

## 2. Samples and experimental techniques

A single-crystal wafer of *n*-type Czochralski-grown Si(001) (*n* = 3 × 10^15^ cm^−3^) with a thickness of 350 µm was implanted with ^64^Zn^+^ ions at an energy of 100 keV and an ion dose of 2 × 10^16^ cm^−2^. An ion beam with a current density of 50 nA cm^−2^ and a diameter of 2 mm was scanned over the substrate surface at an angle of 7°. The samples were subsequently pre-annealed in a neutral atmosphere at 673 K and then annealed at 873, 973, 1173 and 1273 K for 60 min in an oxygen atmosphere. HRXRD measurements were performed on a D8 Discover (Bruker AXS) diffractometer equipped with a conventional X-ray tube operating at 1.6 kW with Cu *K*α_1_ radiation. A high-resolution experimental setup was achieved using a Goebel mirror with a fourfold Ge(220) Bartels-type primary beam monochromator and a threefold Ge(220) diffracted beam analyzer. With this configuration, a wavelength dispersion of the primary beam of Δλ/λ ≃ 7 × 10^−5^ and a divergence of only 12′′, both for the primary and for the diffracted beams, were achieved. A qualitative phase analysis of the thin implanted layers was performed in an out-of-plane grazing-incidence geometry (parallel beam configuration).

For a detailed defect structure analysis of ion-implanted layers by HRXRD, it is necessary to separate the coherent and incoherent (diffuse) parts of the scattered intensity from the whole diffraction pattern. The pure coherent component of the scattered intensity can be used for the determination of a strain profile and the distribution of the static Debye–Waller factor. The diffuse X-ray scattering can be used for defect structure analysis, namely, for estimating the concentration of defects and their size. Moreover, the two-dimensional shape of the diffuse scattering is used for determination of the deformation field symmetry caused by a defect.

The original procedure for reciprocal space map (RSM) analysis (Shalimov *et al.*, 2007 ▶) is based on the following assumptions of diffracted intensity distribution along the *q_x_* axis:

(i) The coherent peak has a bell-like shape, meaning that there is no scattering from mosaic structure or grains.

(ii) The full width at half-maximum (FWHM) of a diffuse peak is wider (two times or more) than one for a coherent peak. The FWHM of the coherent peak should be comparable to the width of the diffractometer instrumental function (∼12′′) and should be practically the same along the *q_z_* direction.

(iii) The intensity of coherent scattering, *I*_coh_, decreases as *I* ≃ 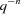 with *n* > 5. The intensity of diffuse scattering *I*_dif_ decreases much slower, 1 ≤ *n* ≤ 4.

From the assumptions, it follows that each *q_x_* intersection of an RSM can be presented as a sum of two bell-like functions with different speeds of decreasing intensity:
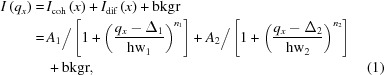
where *A_i_* is the maximal intensity of the peak (*i* = 1, 2), hw_*i*_ is the FWHM of the peak, *n_i_* is the intensity decrease speed, Δ*_i_* is the deviation from *q_x_* = 0 for the coherent and diffuse components, respectively, and bkgr is a constant component of intensity caused by detector noise *etc*.

In the present work, the samples were oriented such that the *q_z_* direction was parallel to the surface normal, so the value of parameter Δ_1_ = 0. In this case the start value of Δ_1_ will be a linear function of *q_z_*. Separation of coherent and diffuse components from experimentally obtained RSMs requires the consecutive analysis of each *q_x_* section at different *q_z_* positions using an optimization procedure. The quality of the curve fitted to an experimental diffraction peak was estimated *via* minimization of the χ^2^-like function
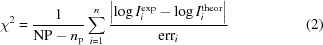
where NP is the number of points at an intersection, *n*_p_ is the number of varied parameters, *I_i_* ^exp^ and *I_i_* ^theor^ are the values of the experimental and calculated intensities at point *i*, and err*_i_* is the relative error of the intensity at each measured point. In formula (2), the real error for each point is present, thus the informative significance of each point can be considered. It is evident that points with large error values have a weak influence on the found parameters.

The ‘pure’ coherent part was fitted using an original procedure (Shcherbachev *et al.*, 2003 ▶), based on a genetic algorithm, to obtain a damage profile which can be described by both strain ∊*_zz_*(*z*) and static Debye–Waller factor *L*_*H*_(*z*) profiles. The value *L*_*H*_ = 8[π(sinθ_B_)/λ]^2^〈*u* ^2^〉 (where θ_B_ is the Bragg angle) is proportional to the mean square displacements of atoms from their sites in the crystal lattice 〈*u* ^2^〉 and can be considered as a characteristic of the degree of crystal imperfection due to amorphization or clustering of point defects. The Fourier components of polarizability in a distorted crystal can be rewritten as 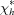 = χ*_h_*exp(−*L*_*H*_). Since 0 ≤ exp(−*L*_*H*_) ≤ 1, introducing the static Debye–Waller factor leads to a decrease in reflectivity of the damaged layer and, thus, to a decrease of diffracted wave amplitude proportional to |
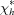
|. Simulation of the diffraction curves was performed in the framework of the dynamical theory of X-ray scattering using the formalism suggested by Wie *et al.* (1986 ▶).

SIMS was used for analyses on a CAMECA IMS-4f instrument with a primary 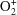-ion current of 150 nA and an energy of 10 keV. The mass spectral resolution was 4000. The focused primary beam was rastered over a 200 × 200 µm area, with detection of ions from an area of 60 µm diameter at the raster center. The sputtering rate was approximately 0.5 nm s^−1^.

Defect concentration depth profiles were obtained from RBS spectra of He^+^ ions in a van de Graaff accelerator with 2 MeV energy using an ion channelling technique (Ziegler, 1972 ▶).

## 3. Results and discussion

**Figure 1:**
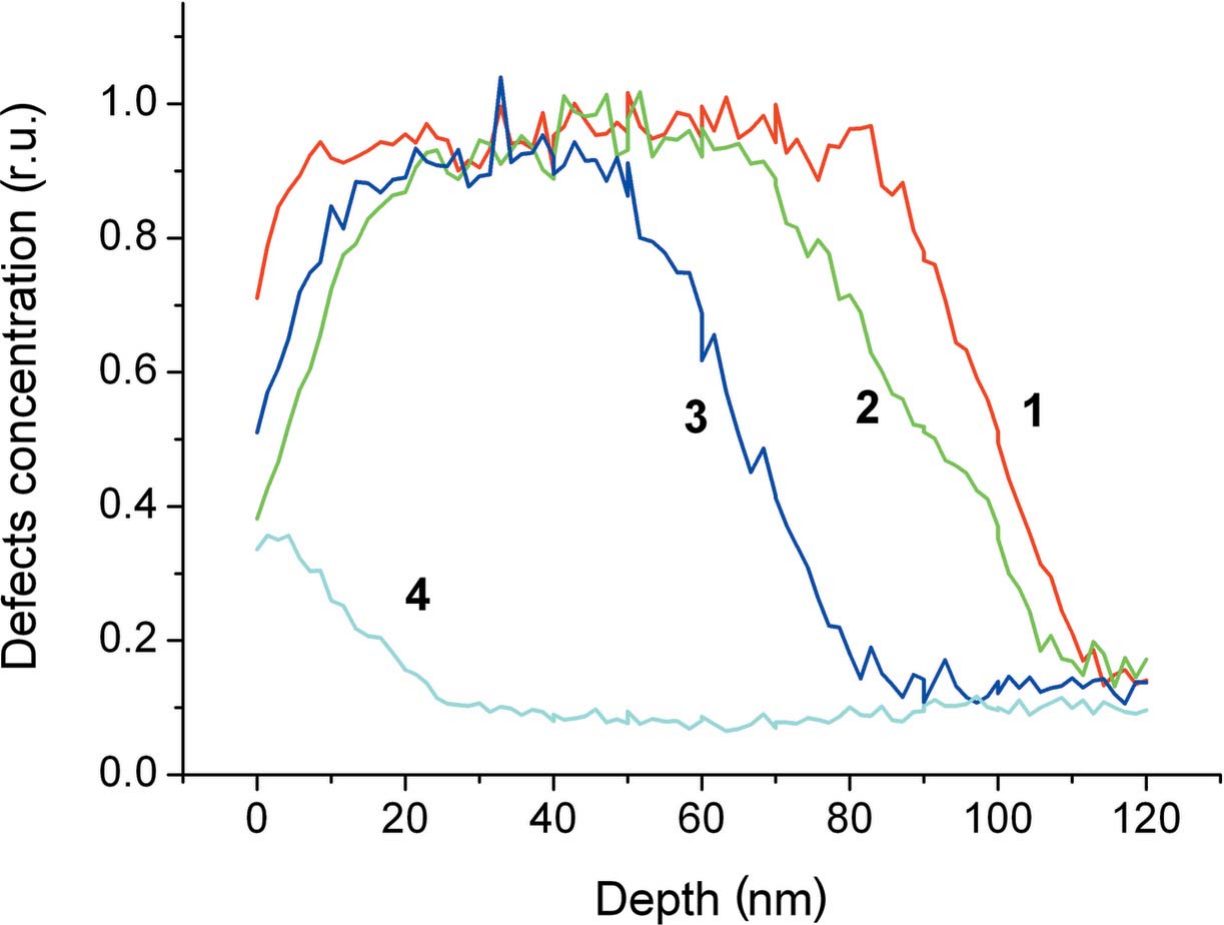
Relative concentration of displaced lattice atoms *versus* depth for the samples implanted with ^64^Zn^+^ ions at an energy of 100 keV and an ion dose of 2 × 10^16^ cm^−2^: curve 1 is for the as-implanted sample; curves 2, 3 and 4 correspond to samples subsequently annealed at 873, 973 and 1173 K, respectively.

**Figure 2:**
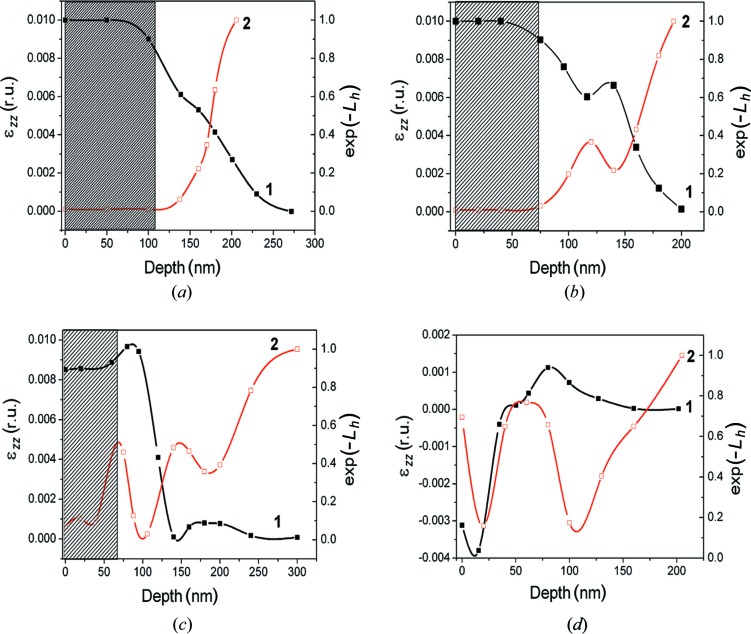
Strain ∊_*zz*_(*z*) (curve 1) and static Debye–Waller factor exp(−*L*_*H*_)(*z*) (curve 2) depth profiles. (*a*) As implanted, and (*b*) annealed at 873 K, (*c*) 973 K and (*d*) 1173 K. The black curve is the strain profile; the red curve is a factor of the exp(−*L_H_*)(*z*) depth profile. The hatched area in (*a*)–(*c*) corresponds to the amorphous layer in accordance with the RBS data.

**Figure 3:**
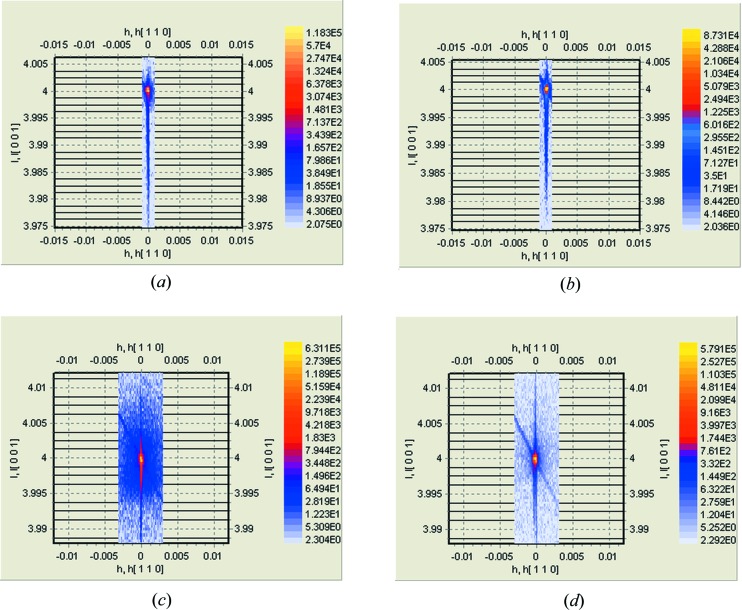
Symmetric Si(004) RSMs measured for the as-implanted sample (*a*), and samples annealed at 873 K (*b*), 973 K (*c*) and 1173 K (*d*). The increase of XRDS around the reciprocal-lattice point after annealing at 973 K can be caused by clusterization of radiation-induced point defects and implanted Zn atoms.

**Figure 4:**
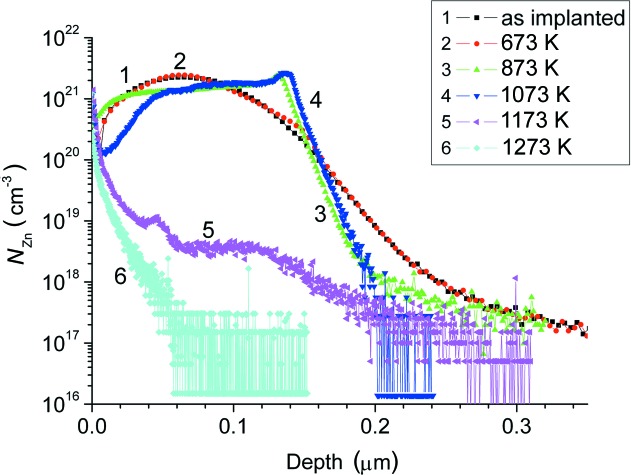
Zn depth profile measured by SIMS for the samples implanted with ^64^Zn^+^ ions at an energy of 100 keV and an ion dose of 2 × 10^16^ cm^−2^.

In the as-implanted state, the surface of the sample is fully amorphized owing to the high-dose implantation. According to a *SUSPRE* (http://www.surrey.ac.uk/ati/ibc/research/modelling_simulation/suspre.htm) simulation, the thickness of the amorphous layer is 100 nm. This is confirmed by the RBS data (Fig. 1 ▶, curve 1). The thickness value of the amorphous layer was used in the fitting of high-resolution diffraction curves to obtain strain and Debye–Waller depth distributions (Fig. 2 ▶ *a*). The amorphous layer is invisible for the setup used in the HRXRD experiment. The thickness of the layer cannot be determined from the fitting of a diffraction curve directly. X-ray diffuse scattering (XRDS) was not observed in an Si(004) RSM (Fig. 3 ▶ *a*). The map has an asymmetric shape along the [00*L*] direction and looks like a map from a substrate with a strained top layer. A part of the damaged layer below an amorphous/crystalline (a/c) interface is enriched by radiation-induced defects, which are a source of tensile strain. The depth distribution of Zn atoms (Fig. 4 ▶), measured by SIMS, has a bell-like shape that was well predicted by the *SUSPRE* simulations.

After 873 K annealing, the thickness of the amorphous part of the damaged layer decreased (Fig. 1 ▶, curve 2). The thickness of the crystalline part of the damaged layer is also decreased, as a result of annealing of radiation-induced point defects and diffusion of Si_i_ interstitials inside the undamaged substrate (Fig. 2 ▶ *b*). The strain and Debye–Waller depth profiles have a peculiarity at a depth of about 150 nm, testifying to cluster formation by interstitial-type defects. SIMS shows a weak segregation of Zn atoms at the same depth (Fig. 4 ▶, curve 2). This area is enriched by so-called end-of-range (EOR) defects that form a band which is some 10–30 nm wide in the implant direction. This band is slightly deeper than the a/c interface. These defects represent the dislocation loops and small clusters of intrinsic radiation-induced interstitial defects (Claverie *et al.*, 1995 ▶;), which are not annealed up to 973 K. However, the XRDS from these defects was not revealed from the RSMs (Fig. 3 ▶ *b*).

Recrystallization of the amorphized layer continued after 973 K annealing (Fig. 1 ▶, curve 3). The strain diminished owing to annealing of radiation-induced defects (Fig. 2 ▶, curve 2). However, the thickness of the layer enriched by interstitial-type defects increased owing to diffusion of interstitial atoms inside the undamaged substrate. At the same time, XRDS from point defect clusters (or dislocation loops) is clearly visible in the RSM (Fig. 3 ▶ *c*).

Fig. 4 ▶ shows a SIMS profile for the sample after annealing at 1073 K (curve 4). We can assume that a profile at 973 K must be somewhere in between. A migration of Zn atoms towards the surface is observed. Reconstruction of the radiation-induced damage layer has occurred from the intrinsic substrate and, apparently, most of the defects have diffused deep into the substrate. The surface is an effective sink for Zn atoms (*N*_Zn_ ≃ 10^21^ cm^−3^ according to SIMS). At a depth greater than 150 nm, the radiation-induced point defects in the damaged layer are relaxed through the interaction with Si matrix interstitials and Zn impurity atoms. This may be due to clusterization of radiation-induced point defects and implanted Zn atoms.

The behavior of Zn profiles after 873 and 973 K annealing can be explained as follows. The peak at 130 nm of curve 3 and the peak at 140 nm of curve 4 in Fig. 4 ▶ are due to an additional accumulation of Zn at the EOR defects (Zn diffuses deeper at 973 K). These positions are correlated with minima of the exp(−*L*_*H*_) factor in Figs. 2 ▶(*b*) (curve 2) and 2 ▶(*c*) (curve 2). At 873 K, Zn has diffused only to the upper part of the band of the EOR defects so that only a narrow peak at 135 nm is observed (curve 3). Annealing at 973 K makes Zn diffuse deeper so that it is trapped in the whole EOR band. This is compatible with the fact that the peak around 140 nm in curve 4 in Fig. 4 ▶ is broad.

After annealing at 1173 K, a reduction in density of the point defects and the complexes responsible for strain of the matrix crystal lattice is observed (Fig. 1 ▶, curve 4, and Fig. 2 ▶ *d*). Apparently, most of the defects have diffused deep into the undamaged substrate. The XRDS background in the Si(004) RSM became less than in the case of 973 K annealing. The damaged layer is fully recrystallized. The zinc depth profile (Fig. 4 ▶, curve 4) has undergone considerable changes. Integration of the SIMS profile (curve 4) over the analyzed depth shows that the full amount of Zn has decreased by a factor of 50 in comparison with the 973 K annealed samples. This could be caused by a rapid diffusion of Zn atoms towards the sample surface. During high-temperature annealing (1273 K), Zn atoms can leave the free top surface, which leads to a decrease of Zn content in the near-surface area (Fig. 4 ▶, curve 5).

Some of the Zn atoms are gathered in the subsurface layer at a depth of 0–40 nm (Fig. 4 ▶, curve 4), which was enriched by Si vacancies (V_Si_). The out-diffusion of Zn from Si is known (Giese *et al.*, 1998 ▶) to proceed mainly *via* the dissociative mechanism Zn_i_ + V_Si_ ↔ Zn_Si_ + E, where E represents an empty interstitial site. Thus, the excess of V_Si_ stimulates Zn diffusion. The source of these vacancies could be both defect complexes containing vacancies and the a/c interface generating V_Si_ defects during recrystallization. Fig. 2 ▶(*d*) shows that the strain value is negative in this near-surface area and the crystal lattice is disordered [a low value of exp(−*L_H_)* factor]. At a depth of 80–110 nm, the content of Zn atoms is about 3–4 × 10^18^ cm^−3^, which is much higher than the limiting equilibrium concentration of Zn in Si, namely 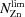 = 6 × 10^16^ cm^−3^. It is safe to assume that Zn precipitation from Si(Zn) solid solution occurs in this sample area. This high-temperature annealing contributes to further growth of clusters that contain Zn. The fact that the growth of Zn-containing clusters is not accompanied by an increase in XRDS may be a result of the formation of Zn-containing phases, which are incoherent with the Si crystal matrix. The strain at depths of greater than 120 nm is determined by weakly associated intrinsic interstitials Si_i_ and Zn atoms that produce the residual tensile strain.

**Figure 5:**
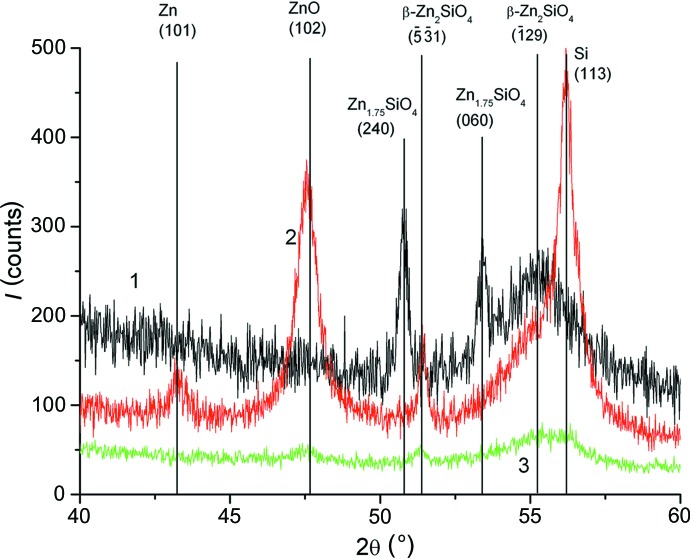
XRD diffraction pattern measured in out-of-plane grazing-incidence diffraction geometry (incidence angle is equal to 0.3°) for the samples annealed at 973 (curve 1), 1173 (curve 2) and 1273 K (curve 3). A scale factor of two is used to avoid spectra overlapping in the plot.

To get more information about the Zn-containing phases, XRD measurements in an out-of-plane grazing-incidence geometry were performed. Fig. 5 ▶ shows the XRD patterns for the samples studied in the angular range 2θ between 40 and 60°. The pattern intensity is scaled up for the sake of clarity. The reference peak positions for Zn-containing phases are also shown as straight lines. This experimental setup allows us to reveal a weak signal from the small number of Zn-containing phases. In the sample annealed at 973 K, peaks observed in the 2θ range 50–56° belong to zinc silicate phases, which cannot be identified exactly owing to the large peak width and low crystallographic symmetry of these phases. After annealing at 1173 K, peaks belonging to zinc, zincate and zinc silicates are present in the diffraction pattern. Annealing at 1273 K does not dissolve all Zn-containing phases. Weak peaks belonging to zinc silicates and zincate phases are still present in the diffraction pattern.

A similar set of Zn-containing phases was observed in SiO_2_ heavily doped with Zn^+^ ions and annealed in an oxygen atmosphere at temperatures up to 1173 K (Amekura *et al.*, 2006 ▶). It was found that ZnO forms after 1 h of annealing at 873 K and disappears after 1173 K annealing. However, in our case, zinc, ZnO and zinc silicates are present even after 1173 K annealing. This state could be the result of an interaction of the following factors: (1) recrystallization of the amorphous part of the layer damaged by implantation; (2) spatial redistribution of Zn atoms due to their migration to the surface; and (3) a diffusion stream of oxygen atoms from the surface deep into the subsurface area. Annealing at 1273 K leads to loss of Zn atoms from the free surface. At this temperature, ZnO can chemically interact with Si and O, which leads to the formation of stable zinc silicate phases.

## 4. Conclusion

A combination of HRXRD, grazing-incidence XRD, RBS and SIMS methods were used to characterize structural transformations of the damaged layer in Si(001) substrates heavily doped by Zn ions after a multistage thermal treatment. To reconstruct strain and Debye–Waller depth profiles from the shape of a diffraction curve, information about the thickness of the amorphous layer from RBS spectra is essential. The shape of SIMS profiles for Zn atoms correlates with the crystal structure of the damaged layer and depends on the presence of factors influencing the mobility of Zn atoms. These factors could be (i) an amorphous/crystalline interface; (ii) EOR defects, which are located slightly deeper than the amorphous/crystalline interface; (iii) the area enriched by V_Si_ defects generated at the amorphous/crystalline interface on annealing; and (iv) a chemical interaction of Zn with Si that leads to formation of Zn-containing phases in the surface layer.
